# Editorial: Advancements in immune heterogeneity in inflammatory diseases and cancer: new targets, mechanisms, and strategies

**DOI:** 10.3389/fmolb.2026.1794468

**Published:** 2026-02-02

**Authors:** Yongqiang Zhang, Yuqin Tang

**Affiliations:** 1 Henan Academy of Innovations in Medical Science, Zhengzhou, China; 2 Clinical Bioinformatics Experimental Center, Henan Provincial People’s Hospital, Zhengzhou University, Zhengzhou, China

**Keywords:** cancer, immune heterogeneity, inflammatory diseaes, new target, therapeutic advancements

Immune homeostasis is essential for maintaining immune balance and host defense, while its disruption underlies a broad spectrum of chronic inflammatory diseases and cancers (McInnes and Gravallese, 2021). Increasing evidence indicates that cellular and molecular immune heterogeneity within inflammatory and tumor microenvironments critically shapes disease progression and therapeutic response (Hou et al., 2021). Although advances in single-cell and spatial multi-omics technologies have revealed diverse immune cell states and dynamic remodeling of the immune landscape, clinical translation remains limited (Guo et al., 2024). Heterogeneous responses to immune checkpoint blockade (ICB) and targeted therapies, as observed in immunologically “cold” tumors such as pancreatic cancer or in therapy-resistant inflammatory conditions, underscore the need for a deeper understanding of immune heterogeneity (Tang et al., 2022; Anandhan et al., 2024; Wu et al., 2024). Therefore, this Research Topic aims to elucidate the dynamic drivers and functional implications of immune heterogeneity in inflammation and cancer, and to promote the development of predictive biomarkers and rational immune-based therapeutic strategies.

Growing evidence indicates that immune heterogeneity is not merely a descriptive feature but an active driver of therapeutic resistance and clinical divergence in inflammatory diseases and cancer (Wang et al., 2021). Therefore, dissecting the mechanisms underlying immune phenotype diversification is essential for understanding why patients with similar pathological diagnoses often exhibit markedly different treatment responses. In this context, several studies provide mechanistic insights into how immune heterogeneity is generated and sustained. In a comprehensive review of lung adenocarcinoma-squamous transformation (AST), Xu et al. illustrate how key signaling pathways, including JAK-STAT, IL-17, and LSD1, promote lineage plasticity and remodel the tumor immune microenvironment, ultimately contributing to resistance against EGFR-targeted therapies and immune checkpoint blockade. Geng et al. summarize the anti-inflammatory role of PSTPIP2 in disorders such as chronic multifocal osteomyelitis and diabetic obesity, demonstrating its ability to suppress IL-1β release and regulate macrophage polarization toward an anti-inflammatory (M2) phenotype. In hepatocellular carcinoma, Qin et al. elucidate through bioinformatics analysis that activation of the RPA1-ETAA1 axis promotes nuclear accumulation of PD-L1, thereby reshaping the immune microenvironment and facilitating immune escape and metastasis, positioning this axis as a potential therapeutic target. Xie et al. demonstrate that the inflammation-to-cancer transition from oral leukoplakia to oral squamous cell carcinoma is driven by the coordinated upregulation of CD46/TREM1 and downregulation of autophagy markers LC3B and ATG5, involving activation of the PI3K–AKT–mTOR and TNF signaling pathways. Together, these findings highlight upstream molecular regulators that actively drive immune landscape diversification in inflammation and cancer.

While mechanistic insights are indispensable, their clinical significance ultimately depends on whether immune heterogeneity can be accurately characterized, quantified, and linked to patient outcomes. Accordingly, several studies employ integrative omics and bioinformatics approaches to construct multi-scale immune portraits with clinical implications. Wang et al. demonstrate that PD-L1 expression on CD14^−^CD16^+^ monocytes is associated with early tuberculosis progression in thymoma patients, suggesting a previously underappreciated immune-mediated vulnerability. In lung adenocarcinoma, Dai et al. identify CD2AP as a poor prognostic marker correlated with enhanced monocyte/macrophage infiltration. Additionally, Cai et al. establish an E2F activity-based gene signature that effectively stratifies high-risk neuroblastoma patients and reveals distinct immune profiles with potential implications for immunotherapy response. These efforts show that immune heterogeneity can be resolved into quantifiable cellular subsets and molecular signatures that are closely linked to disease progression and therapeutic outcomes.

Despite increasingly refined immune characterization, translating immune heterogeneity into actionable therapeutic strategies remains a central challenge. Accumulating evidence suggests that immune-based interventions are most effective when tailored to baseline immune states and molecular subtypes, driving growing interest in heterogeneity-informed targets and optimized intervention strategies (Wang et al., 2021). Bartkeviciene et al. disclose that baseline aryl hydrocarbon receptor (AHR) expression in peripheral blood mononuclear cells from pancreatic cancer patients significantly influences the immunological effects of different AHR modulators, supporting its potential utility as a biomarker for personalized immunotherapy stratification. Dong et al. and Liu et al. review and discuss the emerging targets, such as JAML and MT1-MMP, and highlight their promise in immune modulation, with evidence suggesting synergistic antitumor effects when JAML agonists are combined with PD-1 blockade. In inflammatory bowel disease, Li et al. show through experimental work that a nano-targeted Gegen Qinlian formulation alleviates ulcerative colitis by promoting macrophage polarization toward an M2 phenotype via the AMPK–PPARγ axis. Together, these translational efforts reinforce the concept that effective immune intervention must proactively account for patient-specific immune contexts.

Looking ahead, research on immune heterogeneity is expected to move toward deeper cross-scale integration and functional translation, with increasing emphasis on connecting immune phenotypes to causal regulatory networks, longitudinal dynamics, and clinically relevant endpoints. Achieving this goal will require tighter coupling between experimental biology, computational modeling, and clinical investigation, particularly to address therapy-induced immune reprogramming and inter-individual variability that remain insufficiently understood. Against this backdrop, the studies highlighted here not only provide a timely synthesis of current advances but also delineate key challenges that motivate continued exploration, underscoring the need for sustained scholarly dialogue to further advance precision immunomodulatory strategies in inflammation and cancer.

In summary, the articles in this Research Topic “*Advancements in Immune Heterogeneity in Inflammatory Diseases and Cancer: New Targets, Mechanisms, and Strategies*” establish an integrated knowledge framework spanning mechanistic discovery, immune landscape characterization, and translational exploration. By linking molecular drivers of immune heterogeneity to clinically actionable biomarkers and intervention strategies, these studies collectively advance the field toward immune landscape-guided precision medicine in inflammation and cancer.
